# Interlaboratory proficiency processing scheme in CSF aliquoting: implementation and assessment based on biomarkers of Alzheimer’s disease

**DOI:** 10.1186/s13195-018-0418-3

**Published:** 2018-08-28

**Authors:** Piotr Lewczuk, Amélie Gaignaux, Olga Kofanova, Natalia Ermann, Fay Betsou, Sebastian Brandner, Barbara Mroczko, Kaj Blennow, Dominik Strapagiel, Silvia Paciotti, Jonathan Vogelgsang, Michael H. Roehrl, Sandra Mendoza, Johannes Kornhuber, Charlotte Teunissen

**Affiliations:** 1 Department of Psychiatry and Psychotherapy, Laboratory for Clinical Neurochemistry and Neurochemical Dementia Diagnostics, Universitätsklinikum Erlangen, and Friedrich-Alexander Universität Erlangen-Nürnberg, Schwabachanlage 6, 91054 Erlangen, Germany; 2 Department of Neurodegeneration Diagnostics, Department of Biochemical Diagnostics, Medical University of Bialystok, University Hospital of Bialystok, Bialystok, Poland; 3 Integrated BioBank of Luxembourg, Dudelange, Luxembourg; 4 Department of Neurosurgery, Universitätsklinikum Erlangen, and Friedrich-Alexander Universität Erlangen-Nürnberg, Erlangen, Germany; 5 Clinical Neurochemistry Laboratory, Sahlgrenska University Hospital, Mölndal, Sweden; 6 Institute of Neuroscience and Physiology, Sahlgrenska Academy at University of Gothenburg, Mölndal, Sweden; 7 Biobank Lab, Department of Molecular Biophysics, Faculty of Biology and Environmental Protection, University of Lodz, Lodz, Poland; 8 BBMRI.pl Consortium, Wroclaw, Poland; 9 Department of Experimental Medicine, University of Perugia, Perugia, Italy; 10 Department of Psychiatry and Psychotherapy, University Medical Center Göttingen (UMG), Göttingen, Germany; 11 Department of Pathology, Memorial Sloan Kettering Cancer Center, New York, NY USA; 12 NYU Center for Biospecimen Research and Development (CBRD), New York, NY USA; 13 Neurochemistry Laboratory and Biobank, Department of Clinical Chemistry, VU University Medical Center, Amsterdam, The Netherlands

**Keywords:** Biobanking, Biomarker, Laboratory standardization, Cerebrospinal fluid, Alzheimer’s disease

## Abstract

**Background:**

In this study, we tested to which extent possible between-center differences in standardized operating procedures (SOPs) for biobanking of cerebrospinal fluid (CSF) samples influence the homogeneity of the resulting aliquots and, consequently, the concentrations of the centrally analyzed selected Alzheimer’s disease biomarkers.

**Methods:**

Proficiency processing samples (PPSs), prepared by pooling of four individual CSF samples, were sent to 10 participating centers, which were asked to perform aliquoting of the PPSs into two secondary aliquots (SAs) under their local SOPs. The resulting SAs were shipped to the central laboratory, where the concentrations of amyloid beta (Aβ) 1–42, pTau181, and albumin were measured in one run with validated routine analytical methods. Total variability of the concentrations, and its within-center and between-center components, were analyzed with hierarchical regression models.

**Results:**

We observed neglectable variability in the concentrations of pTau181 and albumin across the centers and the aliquots. In contrast, the variability of the Aβ1–42 concentrations was much larger (overall coefficient of variation 31%), with 28% of the between-laboratory component and 10% of the within-laboratory (i.e., between-aliquot) component. We identified duration of the preparation of the aliquots and the centrifugation force as two potential confounders influencing within-center variability and biomarker concentrations, respectively.

**Conclusions:**

Proficiency processing schemes provide objective evidence for the most critical preanalytical variables. Standardization of these variables may significantly enhance the quality of the collected biospecimens. Studies utilizing retrospective samples collected under different local SOPs need to consider such differences in the statistical evaluations of the data.

**Electronic supplementary material:**

The online version of this article (10.1186/s13195-018-0418-3) contains supplementary material, which is available to authorized users.

## Background

A growing body of evidence supports application of the cerebrospinal fluid (CSF) biomarkers as diagnostic tools for Alzheimer’s disease (AD) and other neurodegeneration disorders [1, 2]. Due to their physical–chemical properties, some of the AD CSF biomarkers are prone to undesired changes in ex-vivo human body fluid samples. It is known that hydrophobic molecules such as amyloid beta (Aβ) peptides, particularly Aβ1–42, absorb to certain plastic surfaces [3–5], or deteriorate following repeated freezing/thawing cycles [6, 7], leading to artificially reduced concentrations. These phenomena are generally unsystematic and hence uncontrollable; for example, after the third freezing/thawing cycle the concentrations of Aβ1–42 significantly decrease in some individual CSF samples, but increase in other samples [6]. Therefore, carefully designed, consequently applied, and continuously controlled preanalytical sample handling standardized operating procedures (SOPs) are of extreme importance. Another dimension of the problem arises when biobanking and multicenter studies come into play. Very generally spoken, two main scenarios are possible in such studies: either the samples (such as the CSF specimens) are collected, processed, and finally locally analyzed in each of the participating centers of a multicenter project; or, alternatively, they are locally collected, processed, and temporarily stored, until they are subsequently shipped to one central laboratory, where all of the analyses take place. The second scenario, for example, is a typical case for large CSF biomarker discovery and validation studies or clinical trials, in which samples are collected and stored in local repositories, and then sent to one central laboratory. If the samples are collected locally but measured centrally, the intercenter variability of the measurements is by definition eliminated, but the differences across the local collection and processing SOPs need to be critically addressed and controlled for. Certainly, preanalytical bias due to differences in processing methods can be minimized in prospective studies if SOP training along with the material needed for sample processing (like test tubes, puncture needles, syringes) are offered to all of the participants before the beginning of sample collection. However, preanalytical bias is unavoidable in retrospective studies, where already stored samples are used from existing repositories.

The concept of the SOP proficiency schemes has been widely applied in nucleic acid extraction methods from different types of matrices [8], but to our best knowledge it has never been implemented in the context of CSF processing. Hence, in this study we attempted to test to which extent differences in the local biobanking processing SOPs influence (in)homogeneity of the resulting aliquots and, in consequence, as an outcome measure, the concentrations of centrally analyzed selected CSF biomarkers. We included three CSF biomarkers in our scheme—Aβ1–42, pTau181, and albumin, reasoning that Aβ1–42 is considered the most preanalytically sensitive biomarker while pTau181 is regarded as the most robust one of the four core CSF AD biomarkers (the two others being Aβ1–40 and total-Tau). For example, compared to total-Tau, pTau181 is less prone to adhesion to test-tube plastics [3] and shows less alteration of the concentrations following repetitive thawing/refreezing cycles of the sample [6]. Albumin, known to be one of the preanalytically most robust proteins in the CSF [9], was also added to the panel as a reference analyte.

## Methods

### Sample preparation and study protocol

**Fig. 1:**
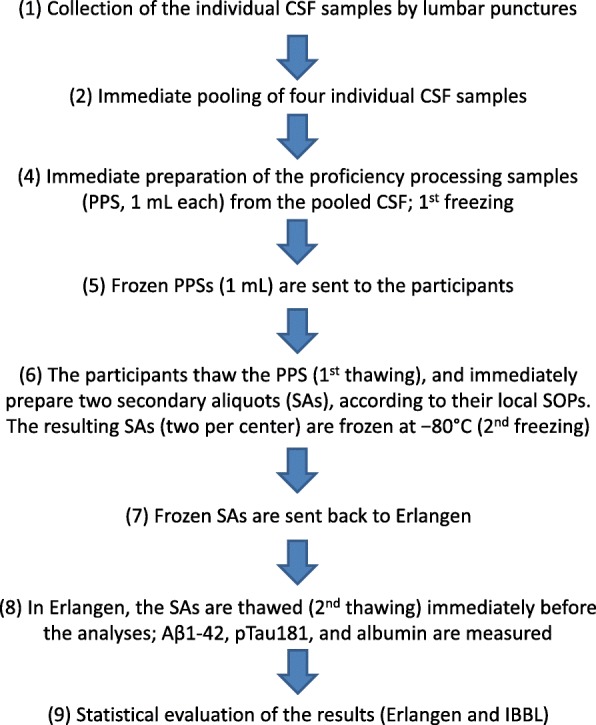
Flow chart of the project. Aβ amyloid beta, CSF cerebrospinal fluid, IBBL Integrated BioBank of Luxembourg, SOP standardized operating procedure

The workflow for the sample preparation is presented in Fig. 1. Briefly, in the Laboratory for Clinical Neurochemistry and Neurochemical Dementia Diagnostics, Erlangen, Germany, CSF from four subjects was pooled, immediately after the lumbar punctures, into one portion of approximately 25 mL, which assured anonymization and nontraceability of the individual samples. This volume was then centrifuged and portioned into 25 primary samples (proficiency processing samples (PPSs)), of 1 mL each, which were immediately frozen at − 80 °C. Ten of these PPSs were then used for homogeneity testing in the Erlangen Laboratory, and 10 PPSs were sent on dry ice to the participating processing laboratories via the logistic unit of the Integrated BioBank of Luxembourg (IBBL). To keep the protocol consistent for all of the participants, the PPSs to be processed by the Erlangen Laboratory also underwent postal circulation in the interlaboratory processing scheme. The participants were asked to thaw the PPSs, and to prepare two secondary aliquots (SAs) strictly according to their local biobanking SOPs; the sole exception was that the resulting SA needed to be 500 μL, irrespective of the volume usually prepared by a participant. The resulting SAs were then frozen according to local procedures, and sent back to the laboratory in Erlangen on dry ice by standard logistics. Each participant was asked to provide the details of the local SOPs via a webpage maintained by the IBBL. The requested information included: storage conditions (temperature and duration) of the PPSs and the resulting SAs, time between thawing of the PPSs and freezing of the resulting SAs, centrifugation data (force, duration, and temperature), and type of secondary storage tubes used.

### Homogeneity testing

Intra-assay variation was tested by 10 repetitions of the measurements of each analyte of interest, and expressed as a corresponding coefficient of variation (CV). Homogeneity testing was performed with the 10 PPSs (1 mL each), stored locally in the Erlangen Laboratory. Briefly, these samples were handled in strictly the same way as the samples sent to the participating laboratories (Fig. 1), with the exception that they were neither sent out nor back (stages 5 and 7 of the protocol were omitted). From each of the 10 PPSs, two SAs (500 μL) were prepared and frozen at − 80 °C until they were assayed, mimicking the workflow for the PPS → SA preparation and the central analyses, followed in the intercenter scheme. Assays for homogeneity testing were those described in the next section.

### Laboratory analyses

The 20 SAs (10 participants × 2 aliquots) were kept at − 80 °C from arrival at Erlangen until the analyses. Aβ1–42 was assayed in duplicate with an ELISA from IBL International (Hamburg, Germany), pTau181 was measured in duplicate with an ELISA from Fujirebio Europe (Ghent, Belgium), and albumin was analyzed with kinetic nephelometry on an Immage 800 nephelometer (Beckman Coulter), following the protocols provided by the vendors. All measurements were run on one ELISA plate (Aβ1–42 and pTau181) or in one analytical run (albumin).

### Statistical analyses

For each analyte of interest, the variability and its components are reported as a set of four statistical metrics: the total unadjusted CV, the within-laboratory coefficient of variation, the between-laboratory coefficient of variation, and the intraclass correlation coefficient (ICC).

For the statistical modeling, the SAs were treated as level-1 units nested within PPSs (level-2 clusters). Mixed-effects variance-components models were used to decompose the total variability of a given analyte into the between-cluster (i.e., random intercept, ψ) and the within-cluster (i.e., residual, θ) variability. ICC, as a metric for the within-cluster agreement, was calculated as ICC = ψ / (ψ + θ). To enable direct comparison of the components of the variance across the three analytes and the two parts of the study, the variance components were normalized for the average concentration of a given analyte (μ):

$$ \mathrm{Between}\hbox{-} \mathrm{center}\ \mathrm{coefficient}\ \mathrm{of}\ \mathrm{variation}\kern0.5em =\kern0.5em \frac{\sqrt{\psi }}{\mu } $$

$$ \mathrm{Within}\hbox{-} \mathrm{center}\ \mathrm{coefficient}\ \mathrm{of}\ \mathrm{variation}=\frac{\sqrt{\theta }}{\mu } $$

Linear regression models were fitted to test whether the between-SA variability of an analyte’s concentrations (defined as the absolute difference of the concentrations of an analyte in the two SAs prepared by a given center divided by the center-specific average of this analyte) depends on the explanatory variables, characterizing the biobanking SOPs of the participants. Mixed-effects models were then fitted to test whether the concentrations of the analytes depend on the explanatory variables, specific for the participants’ SOPs. Pairwise correlations between continuous variables are presented as Spearman’s rank correlation coefficients (ρ). For the hypotheses testing, *p* < 0.05 was considered significant. All analyses were performed with Stata 14.2 (StataCorp, College Station, TX, USA).

## Results

### Homogeneity testing

**Fig. 2:**
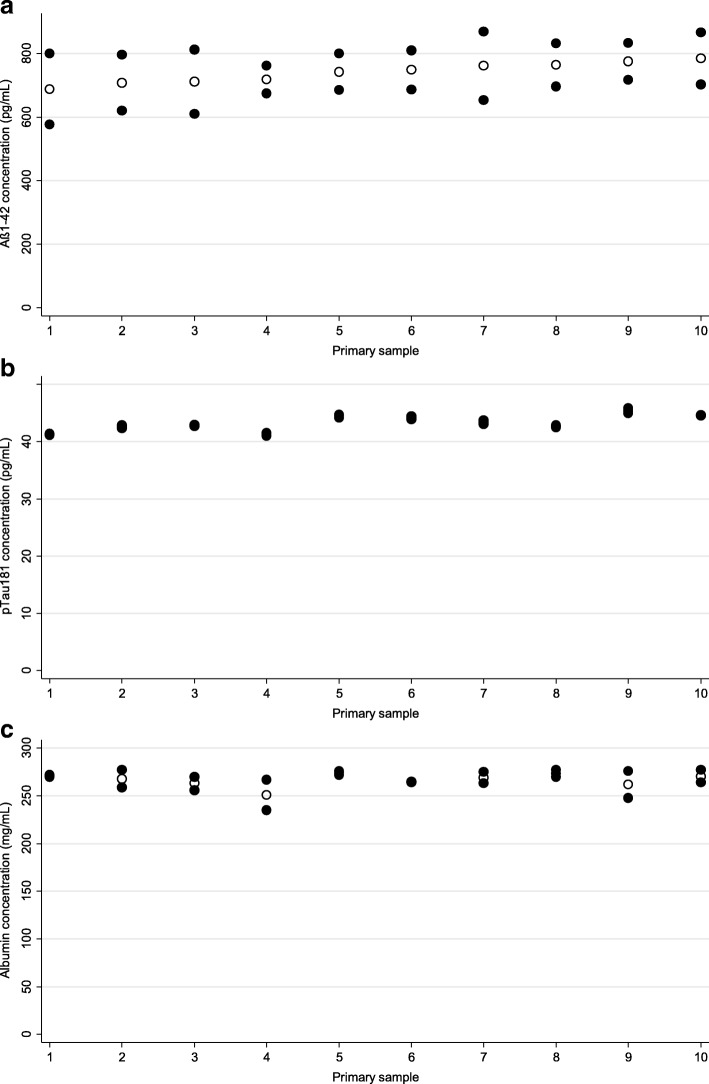
Results of homogeneity testing for Aβ1–42 (**a**), pTau181 (**b**), and albumin (**c**). Individual concentrations obtained in aliquots prepared from 10 primary samples presented as filled circles; averages presented as hollow circles. Aβ amyloid beta

CVs of the intra-assay imprecision of the measurements were 2.9%, 3.9%, and 3.5% for Aβ1–42, pTau181, and albumin, respectively. The results of the homogeneity analyses are presented in Table 1 (left columns) and Fig. 2. The pTau181 and albumin results were characterized by very low overall variability (CV = 3.2% and 4%, respectively), which was comparable to the intra-assay imprecision of the analytical methods used. In the case of Aβ1–42, a CV of 12% was observed, which is considerably higher compared to the method’s intra-assay imprecision. The coefficients of between-cluster (i.e., between-PPS) variation were acceptably low for all three analytes (< 0.1% for Aβ1–42 and albumin, and 3% for pTau181). In contrast, the coefficient of within-PPS variation (i.e., variation between the SAs obtained from a given PPS) of Aβ1–42 (12%) turned out higher than those of pTau181 (0.8%) and albumin (4%). The ICCs of Aβ1–42 and albumin (< 0.01 in both cases) were much lower than the ICC of pTau181 (0.93). In the case of Aβ1–42, a low ICC derives from a relatively high within-cluster (i.e., between-SA) variability compared to the between-cluster (i.e., between-PPS) variability. In the case of albumin, taking into consideration its low total variability (CV = 4%), the low ICC should be treated as a neglectable nuisance.

**Table 1:** Overall coefficients of variation (CVs), parameters of variance-component models decomposing total variability into between-cluster and within-cluster variability, and corresponding intraclass correlation coefficients (ICCs)

Biomarker	Intracenter scheme^a^				Intercenter scheme^b^			
	CV (%)^c^	$\frac{\sqrt{\psi }}{\mu }$ (%)	$\frac{\sqrt{\theta }}{\mu }$ (%)	ICC	CV (%)^c^	$\frac{\sqrt{\psi }}{\mu }$ (%)	$\frac{\sqrt{\theta }}{\mu }$ (%)	ICC
Aβ1–42	12	< 0.1	12	< 0.01	31	28	10	0.89
pTau181	3.2	3	0.8	0.93	8	2.5	7	0.11 (0.88)^d^
Albumin	4	< 0.1	4	< 0.01	10	2	9	0.05 (0.92)^d^

*μ* represents overall average concentration of a given biomarker in a given scheme

*Aβ* amyloid beta, *PPS* proficiency processing sample, *SA* secondary sample

^a^In the intracenter scheme, between-cluster (random intercept) variability (*ψ*) was the variability of the results obtained from 10 PPSs, and within-cluster (residual) variability (*θ*) was the variability of the results obtained in two SAs prepared from each PPS

^b^In the interlaboratory scheme, between-cluster (random intercept) variability (*ψ*) was the variability of the results obtained from 10 PPSs sent to the participating laboratories, and within-cluster (residual) variability (*θ*) was the variability of the results obtained in two SAs prepared in each laboratory from the PPS

^c^Unadjusted total coefficient of variation of the results of the measurements of 20 SAs treated as 20 independent samples, irrespective of their origin from the PPSs

^d^ICCs after exclusion of the two centers (numbers 7 and 8) with apparent failure in their standardized operating procedures

### Interlaboratory processing variability

**Fig. 3:**
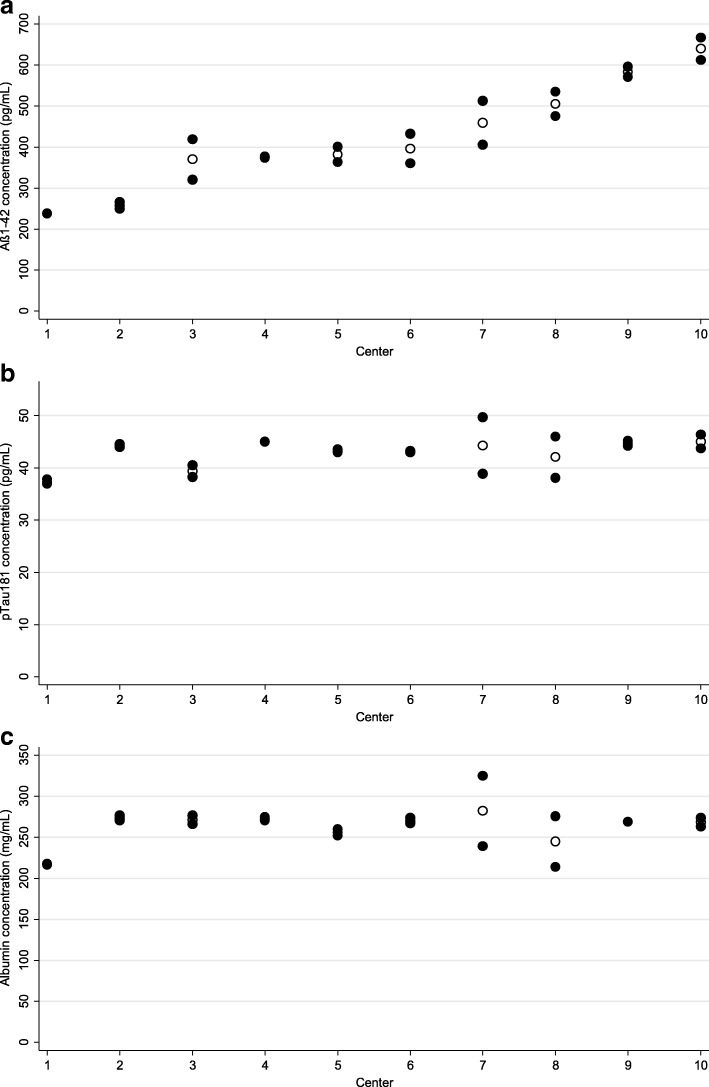
Results of interlaboratory processing scheme, for analytes of interest: Aβ1–42 (**a**), pTau181 (**b**), and albumin (**c**). Concentrations obtained in aliquots prepared by a given laboratory from primary sample presented as filled circles; laboratory-specific averages presented as hollow circles. Aβ amyloid beta

Ten laboratories participated in the intercenter testing; the results of this part of the study are presented in Table 1 (right columns) and Fig. 3. In the case of Aβ1–42, we observed considerably large overall variability (CV = 31%), much larger than in the case of the other two analytes, as well as much larger than the 12% CV of Aβ1–42 in the homogeneity study. The between-center component of this variability was even more evident, with the corresponding coefficient of Aβ1–42 exceeding more than 10 times the coefficients of the other two analytes. In contrast, the coefficients of the within-center variability of all three analytes (10%, 7%, and 9% for Aβ1–42, pTau181, and albumin, respectively) were comparable.

**Fig. 4:**
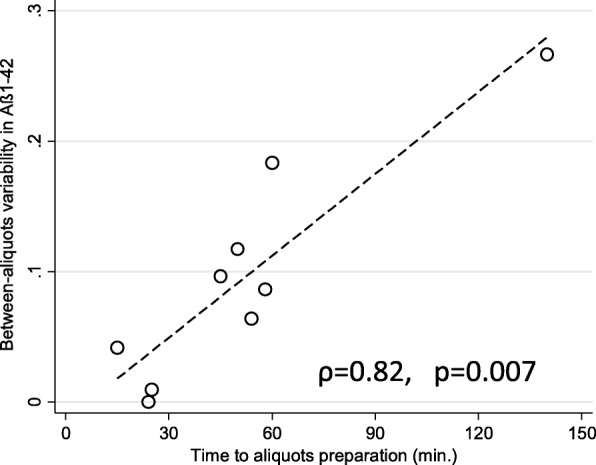
Correlation of variability between Aβ1–42 concentrations measured in two aliquots prepared by a given laboratory and duration of preparation of these aliquots. Variability expressed as absolute difference between concentrations in the two aliquots prepared by a given laboratory divided by average of these two concentrations. Aβ amyloid beta

A reasonably large ICC of Aβ1–42 (0.89) indicates better within-center than between-center agreement between the SAs. Much lower ICCs in the case of pTau181 (0.11) and albumin (0.05) are consequences of large within-center variability in two laboratories (numbers 7 and 8). This contributed significantly to high within-center variability and, correspondingly, to the low ICCs of these two analytes in the whole scheme. After exclusion of the results of these two centers from the statistical analysis, the within-center coefficients of variation of both pTau181 and albumin dropped to 2%, and the ICCs of pTau181 and albumin increased to 0.88 and 0.92, respectively, indicating an excellent within-center agreement.

Additional file 1: Table S1 presents details of the center-specific protocols, considered as potential confounders. Linear regression models were applied to test which of these confounders could explain between-aliquot (i.e., within-center) variability of the Aβ1–42 concentrations. Among the variables tested—storage duration and temperature of the PPSs, force, duration, and temperature of the centrifugation, duration of the preparation of the secondary aliquots, and duration and temperature of the SAs storage at the local biobanks—only the effect of the duration of the preparation of the secondary aliquots turned out to be significant, both unadjusted (*p* < 0.001) and adjusted for other explanatory variables (*p* = 0.042). In particular, the between-aliquot variability of the Aβ1–42 concentrations was not statistically significantly associated with its center-specific average concentration (*p* = 0.76; Additional file 2: Figure S1). Due to a large diversity of the secondary storage tubes used for the aliquoting (practically each participant used a different type of the storage tubes), it was impossible to quantify effects of the biobanking storage tubes. The correlation between the duration of the SAs preparation and the variability in Aβ1–42 concentrations between the SAs in nine laboratories (one participant did not report this metric) is presented in Fig. 4.

Finally, mixed-effects models were fitted to test whether the center-specific confounders affect the concentrations of the individual analytes. For all three of them, the effect of the centrifugation force, unadjusted for other covariates, was positive and either significant (pTau181, *p* = 0.001) or borderline insignificant (Aβ1–42, *p* = 0.087; albumin, *p* = 0.077). The effects of other variables, unadjusted for one another, were insignificant for all three analytes. Interestingly, although all PPSs reached the participants in deeply frozen status, we observed that the lowest concentrations of Aβ1–42, but neither pTau181 nor albumin, were measured in the SAs prepared in the two geographically most-distant centers (numbers 1 and 2, the only two participants from the USA), although the between-aliquot agreement of the results from these two centers was excellent. Further, in one center (number 4), the PPS was erroneously stored at + 4 °C for a prolonged time which, apparently, affected neither the concentrations nor the between-aliquot variability of any of the three analytes. Pairwise correlations of the average concentrations of the three analytes turned out insignificant (*p* > 0.25 for all three pairs after Bonferroni correction for multiple correlations; data not shown).

## Discussion

In this paper, we report the results of a proficiency processing scheme, evaluating variation between aliquots of CSF samples arising from the differences across local biobanking procedures. Whereas we observed neglectable variability in the concentrations of two analytes (albumin and pTau181) across the laboratories and the aliquots, the variability in Aβ1–42 concentrations in the aliquots prepared by the 10 participating laboratories reached 31%. By decomposition of the total variability into within-laboratory and between-laboratory components, we showed that in addition to the variability between aliquots prepared by *different* laboratories, the aliquots prepared *within* a given laboratory can also significantly differ from one another. Finally, we conclude that the duration of the sample processing is probably the most important factor contributing to this variability.

For each analyte of interest, the variability and its components are reported as a set of four statistical metrics: the total unadjusted coefficient of variation, the within-laboratory coefficient of variation, the between-laboratory coefficient of variation, and the intraclass correlation coefficient. The application of coefficients, instead of nonnormalized metrics (like, for example, standard deviations expressed in the units of measurements), enables a direct comparison of the variability and its components for quantities (the concentrations of the analytes), measured on different scales. We believe that such an approach could be also applied for other proficiency testing schemes, irrespective of the analytes tested, since it provides the most comprehensive way to interpret the results. Ideally, the CV, the within-laboratory and the between-laboratory coefficients of variation should be as close as possible to 0, but with the between-laboratory coefficient higher than the within-laboratory coefficient, which would result, in an ideal case, in the ICC as close as possible to 1. The higher the CV, the larger the total variability of the results, and if a CV exceeds some triggering threshold level (which perhaps should be defined taking into consideration factors such as the measurement’s method imprecision) the total variability should be decomposed and analyzed closer. In contrast, in cases with a low overall CV, it does not make much sense, we believe, to analyze the components of the variability in more detail. For example, in this study, the within-PPS variability (i.e., the variability between two aliquots obtained from a given primary sample) of albumin in the intralaboratory part is several fold larger (4%) compared to its between-PPS component (< 0.1%). As a matter of fact, the whole variability of the albumin’s concentration seems to result exclusively from its between-aliquot component, which, in turn, causes seemingly a very poor agreement between the aliquots (ICC < 0.01). However, considering the overall low variability of the albumin concentrations, this would be an overinterpretation; in this particular case it is reasonable to conclude that the different biobanking procedures do not generate significant variability. An entirely different issue is Aβ1–42 in the interlaboratory study, with a very high total CV (31%), much larger compared to the coefficients of the two other analytes in the intercenter study, as well as the coefficients of all three analytes in the intracenter study (≤ 12% for all analytes). In this case, majority of the total variance comes from the between-laboratory component (28%), with a minor part (10%) resulting from the within-laboratory (i.e., between-aliquot) variability. This pattern tells us that the biobanking SOPs are inhomogeneous across the laboratories and, so long as Aβ1–42 is the analyte of interest, the origin of the aliquots from particular repositories has to be taken into account in the statistical analysis. Indeed, if aliquots from centers number 1 and number 10 were sent for a hypothetical biomarker discovery project to a central laboratory, the fact that the samples were prepared under different SOPs would be enough to misinterpret the measurement results as being “normal” (samples from laboratory number 10) or “pathologic” (laboratory number 1), irrespective of the real status of the patients.

Interestingly, pTau181 and albumin showed low total variability (CVs ≤ 10%), but with an unexpected distribution of its components: there was on average much larger discrepancy between the aliquots generated by the same laboratory (7% and 9%) than the discrepancy across the laboratories (≤ 2.5%). Such distribution of the variability components results in a low between-aliquot agreement, as expressed by the low ICCs (0.11 and 0.05). This pattern is brought about by two outlying centers (numbers 7 and 8; Fig. 3) for which the concentrations of pTau181 and albumin *on average* fitted very well to the concentrations in the aliquots prepared by the remaining participants, but with large discrepancies between the particular aliquots. Indeed, exclusion of the results from these two centers reduced the overall within-laboratory variability by a factor of four, and increased the between-aliquot agreement (as expressed by the ICCs) 8–18 times (Table 1).

Both low within-laboratory and between-laboratory variability of the pTau181 and albumin concentrations in this study indicate the homogeneity of the PPSs sent to the participants, and also the preanalytical robustness of these two analytes. Hence, we suggest that CSF biobanks may perhaps consider measurements of pTau181 and/or albumin in a series of their aliquots resulting from one patient’s primary sample as a control measure to test whether the local procedures fulfill homogeneity criteria.

We observed that the duration of the preparation of the secondary aliquots and the centrifugation force are the two major confounders contributing to the between-aliquot variability of Aβ1–42 concentrations, and to the concentrations of the biomarkers, respectively. Although these covariates were identified as major confounding factors influencing biomarker concentrations in other studies [10–13], we feel that it is premature to derive any conclusions on their role as confounders in biobanking protocols before future studies in a similar setting are completed.

This study is not without limitations. One of these is that the primary samples, sent to the participants, were already pretreated before shipment. First, they were prepared from a pool of four individual CSF samples; and, second, they needed to be frozen. Therefore, in this scheme one additional freezing/thawing cycle was applied compared to an everyday situation, in which a locally collected body fluid sample is normally not frozen before further processing. We believe, however, that at least three arguments justify the procedure as it was applied in our study: first, two freezing/thawing cycles do not bring about more variability in the concentrations of the CSF AD biomarkers than one cycle does [6, 7]; second, certain large-scale projects apply an intermediate freezing/thawing cycle before the aliquots are eventually stored in a biobank [14]; and, third (and perhaps crucial), it is not possible, in schemes like this one, to reduce the number of the freezing/thawing cycles to one, if processing items (samples) are supposed to reach distant laboratories in the most standardized conditions.

Finally, considering that this is probably the first study of this kind, we do not think we could give any kind of detailed recommendations regarding the between-center variability acceptance criteria or ways to improve the CSF biobanking SOPs. We may only speculate that future acceptance criteria should consider at least precision of the analytical methods and the values of the clinically relevant critical concentrations. The former issue is of pure statistical matter, and might be achieved by further decomposition of the total variability by introduction of one additional level in the hierarchical regression models, leading to the intra-assay imprecision (f.e., between-duplicate variability, L1) nested within secondary aliquots (L2) nested within centers (L3). The latter issue is much more complex, as it needs to consider which extent of error, particularly around the biomarkers’ diagnosis-relevant decision levels (laboratory cutoff values), is acceptable in a given study. Similarly, in this single study the centrifugation force and the duration of the preparation of the secondary aliquots seem of relevance for the biobanking quality, but we believe that further studies are warranted to confirm these observations.

## Conclusions

We believe that proficiency processing schemes, like these reported in the literature [8] as well as the one presented here, provide objective evidence for the most critical preanalytical variables. Standardization of these variables may significantly enhance the quality of the prospectively collected biospecimens and prevent from misinterpretations of the results from the retrospectively collected samples. For example, in our study the duration of the preparation of the aliquots from a primary CSF sample seems to be the most critical variable affecting the *within-laboratory* Aβ1–42 variability. As for the *between-laboratory* variability, centrifugation conditions appear to be a critical factor; however, further studies with a larger number of participants are necessary to confirm this finding. In the future, other confounders also need to be addressed; for example, the type of pipette tips and the technique of how a primary sample is pipetted to prepare secondary aliquots may definitely contribute to the intercenter inhomogeneity. Finally, the higher the number of participating laboratories in further schemes, the more reliable will be the elucidation of the impact of the most critical processing variables on analytes of interest [15]. For this reason, proficiency processing schemes are needed to support development of preanalytical CEN/ISO standards (http://www.spidia.eu/).

## Additional files

Additional file 1: **Table S1.** Details of the SOPs to prepare secondary aliquots (SAs) reported by the 10 participants. (DOCX 16 kb)

Additional file 2: **Figure S1.** Bland–Altman plot of differences between Aβ1–42 concentrations in two aliquots prepared by each participating center as a function of the center-specific average of Aβ1–42 concentrations. (PDF 86 kb)

## Abbreviations

AD: Alzheimer’s disease
Aβ: Amyloid beta
CSF: Cerebrospinal fluid
CV: Coefficient of variation
ICC: Intraclass coefficient
ISO: International Standardization Organization
PPS: Proficiency processing sample
SA: Secondary sample
SOP: Standardized operating procedure

## References

[1] Lewczuk P, Riederer P, O'Bryant SE, Verbeek MM, Dubois B, Visser PJ, Jellinger KA, Engelborghs S, Ramirez A, Parnetti L (2018). Cerebrospinal fluid and blood biomarkers for neurodegenerative dementias: an update of the Consensus of the Task Force on Biological Markers in Psychiatry of the World Federation of Societies of Biological Psychiatry. World J Biol Psychiatry.

[2] Dubois B, Feldman HH, Jacova C, Hampel H, Molinuevo JL, Blennow K, DeKosky ST, Gauthier S, Selkoe D, Bateman R (2014). Advancing research diagnostic criteria for Alzheimer’s disease: the IWG-2 criteria. Lancet Neurol.

[3] Lewczuk P, Beck G, Esselmann H, Bruckmoser R, Zimmermann R, Fiszer M, Bibl M, Maler JM, Kornhuber J, Wiltfang J (2006). Effect of sample collection tubes on cerebrospinal fluid concentrations of tau proteins and amyloid beta peptides. Clin Chem.

[4] Perret-Liaudet A, Pelpel M, Tholance Y, Dumont B, Vanderstichele H, Zorzi W, Elmoualij B, Schraen S, Moreaud O, Gabelle A (2012). Risk of Alzheimer's disease biological misdiagnosis linked to cerebrospinal collection tubes. J Alzheimers Dis.

[5] Kofanova OA, Mommaerts K, Betsou F (2015). Tube polypropylene: a neglected critical parameter for protein adsorption during biospecimen storage. Biopreserv Biobank.

[6] Zimmermann R, Lelental N, Ganslandt O, Maler JM, Kornhuber J, Lewczuk P (2011). Preanalytical sample handling and sample stability testing for the neurochemical dementia diagnostics. J Alzheimers Dis.

[7] Vanderstichele H, Van Kerschaver E, Hesse C, Davidsson P, Buyse MA, Andreasen N, Minthon L, Wallin A, Blennow K, Vanmechelen E (2000). Standardization of measurement of beta-amyloid(1-42) in cerebrospinal fluid and plasma. Amyloid.

[8] Gaignaux A, Ashton G, Coppola D, De Souza Y, De Wilde A, Eliason J, Grizzle W, Guadagni F, Gunter E, Koppandi I (2016). A biospecimen proficiency testing program for biobank accreditation: four years of experience. Biopreserv Biobank.

[9] Rosenling T, Stoop MP, Smolinska A, Muilwijk B, Coulier L, Shi S, Dane A, Christin C, Suits F, Horvatovich PL (2011). The impact of delayed storage on the measured proteome and metabolome of human cerebrospinal fluid. Clin Chem.

[10] Schoonenboom NS, Mulder C, Vanderstichele H, Van Elk EJ, Kok A, Van Kamp GJ, Scheltens P, Blankenstein MA (2005). Effects of processing and storage conditions on amyloid beta (1-42) and tau concentrations in cerebrospinal fluid: implications for use in clinical practice. Clin Chem.

[11] Kaiser E, Schonknecht P, Thomann PA, Hunt A, Schroder J (2007). Influence of delayed CSF storage on concentrations of phospho-tau protein (181), total tau protein and beta-amyloid (1-42). Neurosci Lett.

[12] Bjerke M, Portelius E, Minthon L, Wallin A, Anckarsater H, Anckarsater R, Andreasen N, Zetterberg H, Andreasson U, Blennow K. Confounding factors influencing amyloid beta concentration in cerebrospinal fluid. Int J Alzheimers Dis. 2010;2010. 10.4061/2010/986310. 10.4061/2010/986310 PMC2925386 20798852

[13] Leitao MJ, Baldeiras I, Herukka SK, Pikkarainen M, Leinonen V, Simonsen AH, Perret-Liaudet A, Fourier A, Quadrio I, Veiga PM, de Oliveira CR (2015). Chasing the effects of pre-analytical confounders—a multicenter study on CSF-AD biomarkers. Front Neurol.

[14] Shaw LM, Vanderstichele H, Knapik-Czajka M, Clark CM, Aisen PS, Petersen RC, Blennow K, Soares H, Simon A, Lewczuk P (2009). Cerebrospinal fluid biomarker signature in Alzheimer's disease neuroimaging initiative subjects. Ann Neurol.

[15] Betsou F, Bilbao R, Case J, Chuaqui R, Clements JA, De Souza Y, De Wilde A, Geiger J, Grizzle W, Guadagni F, et al. Standard PREanalytical Code version 3.0. Biopreserv Biobank. 2018. 10.1089/bio.2017.0109. 10.1089/bio.2017.0109 PMC11708182 29377712

