# Supplementary material for: Interlaboratory proficiency processing scheme in CSF aliquoting: implementation and assessment based on biomarkers of Alzheimer’s disease
Source: Alzheimers Res Ther. 2018 Aug 28;10:87. doi: 10.1186/s13195-018-0418-3 (PMC6114189; doi:10.1186/s13195-018-0418-3)
Supplement: Supplementary file 1 — Table S1. Details of the SOPs to prepare secondary aliquots (SAs) reported by the 10 participants. (DOCX 16 kb) [file 13195_2018_418_MOESM1_ESM.docx]

Table S1 Details of the SOPs to prepare the secondary Aliquots (SA) as reported by the ten participants.

| Center id | PPS Storage Temp. (°C) | PPS Storage Duration (Days) | Centrifugation data (force, temperature and duration, brake (Y/N)) | PPS => SA Duration (min.) | SA Storage Temp. (°C) | SA Test Tubes |
| --- | --- | --- | --- | --- | --- | --- |
| 1 | -80 | 6 | < 500g / RT / 10-15' / Yes | 24 | -80 | Nalgene 1.5 ml (Ref. 5000-1020) |
| 2 | -80 | 4 | 1000-3000g / 4°C / 10-15' / Yes | 54 | -80 | Eppendorf 1.5 ml (Ref. 22363263) |
| 3 | -80 | 1 | 400g, 10mim, brake 5, 4°C / Yes | 141 | -80 | Matrix tube (0.5 ml cryotube) (Thermo-Fischer  Ref. 3744): |
| 4 | -20 | 0 | 2000xG at 20 degrees, for 10 min / Yes | 25 | -20 | Sarstedt 2 ml (Ref. 72.609.001) |
| 5 | -80 | 5 | 1000-3000g / RT / 10-15' / Yes | 45 | -80 | Sarstedt 0.5 ml (Ref. 72.730.105) |
| 6 | +4 | 27 | 3100g, RT, 10 min / No | 60 | -80 | Eppendorf 1.5 ml (Ref. 22363263) |
| 7 | -80 | 2 | 1000-3000g / RT / 10-15'; Brake: 4, 20C, 10 min., 1800 g / Yes |  | -80 | Sarstedt 1.5ml (72.703) with screw cap Sarstedt (72.703) orange |
| 8 | -80 | 7 | 1000-3000g / RT / 10-15':Break strength 3/10 / Yes | 50 | -80 | Micronic 0.5 ml |
| 9 | -80 | 1 | 1000-3000g / RT / 10-15' / Yes | 15 | -80 | Matrix tube (0.5 ml cryotube) (Thermo-Fischer Ref. 3744) |
| 10 | -80 | 1 | 1000-3000g / RT / 10-15' / No | 58 | -80 | Other |
