# Supplementary figures and images for: Interlaboratory proficiency processing scheme in CSF aliquoting: implementation and assessment based on biomarkers of Alzheimer’s disease
Source: Alzheimers Res Ther. 2018 Aug 28;10:87. doi: 10.1186/s13195-018-0418-3 (PMC6114189; doi:10.1186/s13195-018-0418-3)

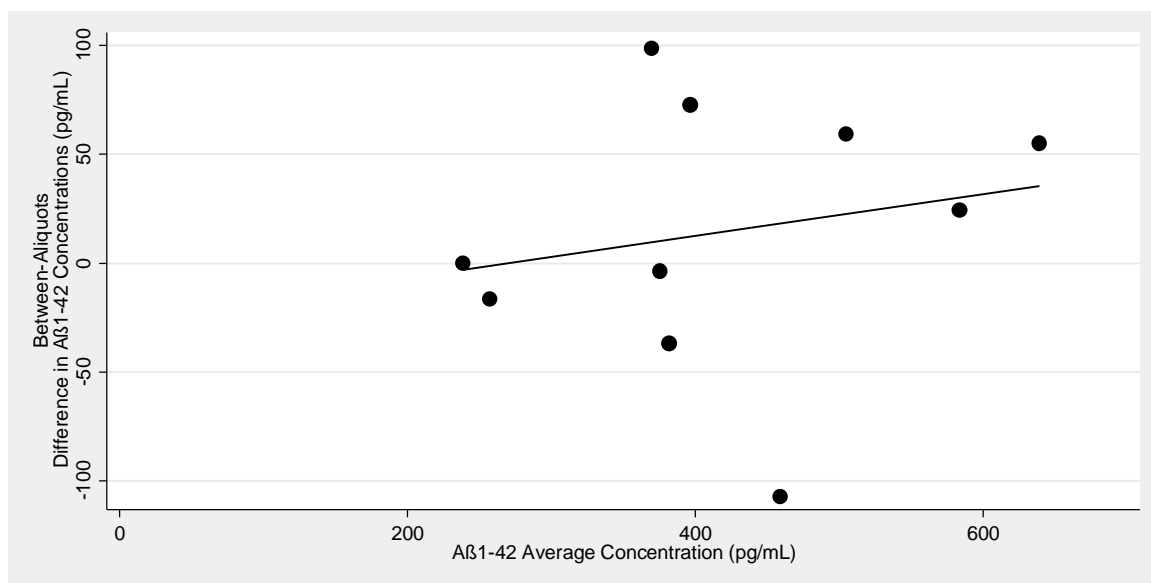

Lewczuk et al, Figure S1

Supplement: Supplementary file 2 — Figure S1. Bland–Altman plot of differences between Aβ1–42 concentrations in two aliquots prepared by each participating center as a function of the center-specific average of Aβ1–42 concentrations. (PDF 86 kb) [file 13195_2018_418_MOESM2_ESM.pdf]
